# Epigenetic Mechanisms Leading to Overexpression of HMGA Proteins in Human Pituitary Adenomas

**DOI:** 10.3389/fmed.2015.00039

**Published:** 2015-06-08

**Authors:** Daniela D’Angelo, Francesco Esposito, Alfredo Fusco

**Affiliations:** ^1^Dipartimento di Medicina Molecolare e Biotecnologie Mediche, Istituto per l’Endocrinologia e l’Oncologia Sperimentale del CNR, Università degli Studi di Napoli “Federico II”, Naples, Italy; ^2^Instituto Nacional de Câncer – INCA, Rio de Janeiro, Rio de Janeiro, Brazil

**Keywords:** HMGA proteins, pituitary tumors, microRNAs, long non-coding RNA, pseudogenes

## Abstract

Overexpression of the high-mobility group A (HMGA)1 and HMGA2 proteins is a feature of all human pituitary adenoma (PAs) subtypes. However, amplification and/or rearrangement of the HMGA2 have been described in human prolactinomas, but rarely in other pituitary subtypes, and no genomic amplification of HMGA1 was detected in PAs. Here, we summarize the functional role of HMGA proteins in pituitary tumorigenesis and the epigenetic mechanisms contributing to HMGA overexpression in these tumors focusing on recent studies indicating a critical role of non-coding RNAs in modulating HMGA protein levels.

## Introduction

Pituitary tumors account for up to 25% of all diagnosed human brain tumors and the estimated prevalence rate in the general population is about 17% (1). They can be classified in different ways depending upon their size, radiographic appearance, endocrine function, morphology, and cytogenesis. Based on the invasiveness, they are divided into (a) benign adenomas, representing the majority of pituitary tumors that grow slowly, and rarely go toward the malignant phenotype; (b) invasive adenomas, benign tumors that may spread to bones of the skull or the sinus cavity below the pituitary gland; (c) pituitary carcinomas, which are malignant and spread into other areas of the central nervous system (brain and spinal cord) or outside of the central nervous system. They are extremely rare (2).

According to their size, pituitary adenomas (PAs) are classified into microadenomas (<10 mm in diameter), macroadenomas (>10 mm), and giant adenomas (>40 mm).

On the basis of their endocrine activity, PAs can be distinguished in hormone producing or clinically non-functioning. About one-third of pituitary tumors are not associated with clinical hypersecretory syndromes and they are called non-functioning pituitary adenomas (NFPAs). Hormone-producing PAs release active hormones in excessive amounts into the bloodstream and they are currently classified into five main immunohistochemistry (IHC) types: prolactinoma (PRL, 25–41%) somatotroph adenomas (GH, 10–15%), corticotroph adenomas (ACTH, about 10%), thyrotroph adenomas (TSH, <1%), and gonadotroph adenomas (FSH-LH <1%), which can be monohormonal or plurihormonal, with or without signs of hypersecretion (3).

Pituitary tumorigenesis is generally considered as a model of the multistep process of tumorigenesis, in which molecular alterations represent the initializing event that transforms cells and hormones and/or growth factors promote cell proliferation (1). Activation of oncogenes or loss of tumor-suppressor genes, as well as alteration of cell cycle mediators and epigenetic modifications, have been frequently reported to be involved in the pathogenesis of PAs (4).

The great majority of PAs arise in a sporadic manner, and only a minority of them is part of hereditary or familial syndromes. The majority of adenomas arising in hereditary syndromes are GH-secreting adenomas and they are associated to MEN-1 (linked to somatic mutations of the tumor-suppressor gene MEN-1), Carney’s complex (CNC) (linked to mutations of the tumor-suppressor gene PRKAR1A), and McCune–Albright syndrome (linked to activating mutations of the *gsp* oncogene). An activating mutation of the *gsp* oncogene is the most frequent genetic alteration in sporadic tumors. It is a point mutation located in the alpha subunit of the G-protein (GNAS), a stimulatory protein of adenylylcyclase at the membrane level (5).

Pituitary tumor transforming gene (PTTG, also known as securin) has been also implicated in pituitary tumorigenesis. PTTG is largely expressed in functional human adenomas but not in normal pituitary tissue (6) and its overexpression in transgenic mice results in pituitary hyperplasia and adenoma formation (7). GADD45γ has been the first identified tumor-suppressor gene in pituitary tumorigenesis. GADD45γ is a negative regulator of cell growth and it is not expressed in the majority of functional and NFPAs (8). In addition, several animal models of cell cycle regulators, such as pRB, CDKs, or CKIs, have demonstrated that alterations of genes involved in cell cycle control are associated with the development of PAs (9). Indeed, mice carrying genetically modified alleles of the retinoblastoma (Rb) tumor-suppressor gene, causing an increase in the E2F1 activity, showed high predisposition to develop pituitary tumors (10), and this occurs also in mice with impaired function of p27 or p18, both converging on pRB pathway (11, 12).

Recently, two different research groups have identified novel somatic mutations in the deubiquitinase gene USP8, all of them harboring in the 14-3-3 protein binding motif and its nearby region, in about one-third of corticotroph adenomas. These mutations enhanced the deubiquitinase catalytic activity of USP8, thus protecting its numerous targets, such as EGFR, from the lysosomal degradation and thereby leading to an enhanced activity of the EGFR pathway (13, 14).

## HMGA as Driver in the Development of Human Pituitary Adenomas

The high-mobility group A (HMGA) family consists of four proteins, HMGA1a, HMGA1b, HMGA1c, encoded by HMGA1 gene through alternative splicing, and HMGA2, encoded by the homonymous gene. They are the most abundant non-histone chromosomal proteins, also known as “architectural transcriptional factors” since they do not possess a direct transcriptional activity, but alter the chromatin structure through their DNA-binding domains, called “AT hooks” (15), by which they bind the minor groove of DNA in AT-rich sequences regulating, positively or negatively, the transcription of several genes (16–18).

High-mobility group A proteins are expressed at high levels during embryogenesis and at low levels in adult tissues, whereas their expression is abundant in several human malignant neoplasias (19). HMGA proteins have a causal role in cell transformation. Indeed, their blocking by antisense methodologies inhibits retrovirally induced malignant transformation of rat thyroid cells (20) while their enforced overexpression induces a transformed phenotype (21), and transgenic mice overexpressing the HMGA proteins develop several benign or malignant neoplasias (22–24).

The involvement of the *HMGA* genes, *HMGA2* in particular, in benign neoplasias has been widely reported. Rearrangements of the *HMGA2* gene were observed in human benign tumors of mesenchymal origin, in which translocations involving the region 12q13–15 (where *HMGA2* gene is located) have been frequently detected (25, 26).

The first evidences of the role of HMGA2 in pituitary tumorigenesis came from the observation that transgenic mice carrying the *HMGA2* gene under the transcriptional control of the cytomegalovirus promoter developed PAs secreting prolactin and growth hormone (23), more frequently in females than in males. In addition, cytogenetic analysis of human prolactinomas revealed trisomy or tetrasomy of chromosome 12 associated with an amplification of the *HMGA2* locus, which correlated with HMGA2 overexpression (27). Even though genomic amplification of the *HMGA2* gene was rarely found in PAs other than prolactinomas (28), the overexpression of HMGA1 and HMGA2 has been reported in a significant number of PAs (29, 30). Moreover, the level of HMGA2 protein has been found positively correlated with tumor invasion and was significantly higher in grade IV than in grades I, II, and III adenomas. High levels of HMGA2 expression were more frequently detected in macroadenomas than in microadenomas and also significantly correlated with the proliferation marker Ki-67 (31).

The mechanism by which HMGA overexpression induces the development of PAs is the enhancement of the E2F1 activity (32). This mechanism is quite unique; although HMGAs bind to the pRB A/B pocket domain, they do not compete with the E2F1 protein in pRB binding, but displace HDAC1 from pRB/E2F1 complex, resulting in enhanced acetylation of both E2F1 and DNA-associated histones, thereby promoting E2F1 activation. The rescue of the adenomatous phenotype when transgenic mice overexpressing the *HMGA2* gene were mated with *E2F1* knockout mice confirmed the role of the enhanced E2F transcriptional activity in the onset of PAs in HMGA transgenic mice (32). Subsequent studies have demonstrated that HMGA1 and HMGA2 can also affect cell cycle by regulating CCNB2, coding for cyclin B2 (30). Indeed, HMGAs are able to directly regulate this gene at transcriptional level, and, consistently, cyclin B2 overexpression was found in human PAs with a significant correlation with HMGA expression. In addition, other mechanisms could be responsible for the oncogenic role of HMGA in pituitary. Among these, the increased expression of IL2 and its receptor in pituitary cells by HMGA (22), and/or induction of the AP-1 activity by enhancing the expression of the AP-1 members FRA-1 and JunB (33) cannot be excluded. Moreover, the pituitary-specific transcription factor Pit-1 (POU domain, class 1, transcription factor 1) was found overexpressed in PAs from Hmga1b and Hmga2 transgenic mice. It has been also demonstrated that Pit-1 is positively regulated by HMGA protein and its overexpression positively correlates with that of HMGA also in human PAs (34).

## Epigenetic Control of HMGA Protein Levels by Non-Coding RNAs

Overexpression of HMGA1 and HMGA2 proteins is a feature of human PAs, and according to a crucial role of their overexpression in pituitary tumorigenesis, transgenic mice overexpressing either hmga1 or hmga2 develop PAs (24). However, while *HMGA2* gene rearrangement and/or amplification was found in a certain number of PAs (27, 28), no genomic alterations of the HMGA1 locus have been detected in PAs, even though overexpression of both HMGA proteins has been described (30, 35, 36). Therefore, other mechanisms accounting for HMGA overexpression in these tumors might be envisaged. Recent studies indicated that both the *HMGA* genes can be modulated by a novel class of epigenetic regulators, known as non-coding RNAs (ncRNAs).

In the last 15 years, thanks to the advent of bioinformatic approaches examining the human transcriptome, it came up that about 70% of the genome is transcribed but only 2% of the human genome is translated into proteins (37). The remaining large proportion of DNA, initially referred to as “junk DNA,” was found to be transcribed as ncRNAs.

Non-coding RNAs comprise multiple classes of RNA that are not transcribed into proteins but have been shown to regulate transcription, stability, or translation of protein-coding genes (38). They include microRNAs (miRNAs or miRs), tRNAs, rRNAs, small nuclear RNAs (snRNAs), and heterogeneous group of long non-coding RNAs (lncRNAs).

## miRNAs Targeting HMGA Proteins

microRNAs are sncRNA molecules of about 22 nt, which induce gene silencing by suppressing protein synthesis or by mRNA degradation. To date, over 1,000 miRNAs have been identified in humans, which regulate about 60% of mammalian genes (39). Interestingly, each miRNA is able to target multiple mRNA and, in turn, each mRNA can be regulated by several miRNAs cooperatively.

microRNA-mediated repression of target gene occurs by perfect or imperfect complementarity with sequence motifs predominantly found within the 3′ untranslated regions (UTRs) of the target mRNAs (40). Altered expression of miRNAs has been associated with various human diseases, including cancer, in which they can act as “onco-miR” or “tumor-suppressor-miR” (41).

Recent studies indicate a critical role of miRNAs in regulation of HMGA expression. Indeed, reduced expression of let-7 is reported in about 42% of PAs and has been inversely correlated with HMGA2 expression and high-grade tumors (42). Subsequently, it has been demonstrated that miR-15, miR-16, miR-26, miR-196a-2, and let-7a, which target both the HMGA genes, are drastically downregulated in a panel of 41 human PAs of different histotypes, and their expression is inversely correlated with the HMGA expression. Moreover, enforced expression of the HMGA-targeting miRNAs reduces cell growth of GH3, a rat GH/PRL pituitary cell line, supporting their tumor-suppressor role (43). Accordingly, the analysis of a miRNA expression profile of GH adenomas versus normal pituitary has unveiled a set of miRNAs constantly deregulated in somatotroph tumors, comprising miR-326, miR-432, and miR-570, targeting HMGA2, miR-34b, and miR-548c-3p both having HMGA1 and HMGA2 as targets, and miR-326 and miR-603 targeting E2F1. Their downregulation was also found in PRL and gonadotroph adenomas, suggesting that it represents a general event in pituitary tumorigenesis. Subsequent functional studies have confirmed the role of the downregulation of these miRNAs in tumor growth and cell cycle regulation. Indeed, a significant reduction of cell number and, accordingly, an increase in the G1 phase population and a decrease in the S phase were observed after transfection with the HMGA-targeting miRNAs. Finally, an inverse correlation was found between miRNA expression and HMGA1 and HMGA2 protein levels in GH-secreting adenomas, suggesting a possible role of these miRNAs in the HMGA/E2F1 pathway, and thereby in the development of PAs (44). Recently, it has been reported that also miR-23b, which was found downregulated in GH, gonadotroph, and null cell PAs, targets HMGA2, and that its overexpression inhibits cell proliferation arresting cells in the G1 phase of cell cycle (45).

## LncRNAs Regulating HMGA Proteins

Long non-coding RNAs are a class of transcribed RNA molecules ranging in length from 200 nt to ~100kb and lacking protein-coding capability. Unlike mRNAs, which exhibit strong conservation across diverse species, lncRNAs are generally poorly conserved, they are preferentially expressed in tissue-specific manner and they can regulate the transcription, stability, or translation of protein-coding genes by different ways that have not been not fully clarified yet (46).

Among the lncRNAs, pseudogenes are a class of RNA molecules that have lost their coding potential because of premature or delayed stop codons, deletions/insertions, and frameshift mutations that abrogate translation into functional proteins (47, 48).

They are classified in processed, if they were generated by retro-transposition of the corresponding protein-coding mRNA, or unprocessed, if they arise by gene duplication and then acquire mutations making them non-functional. Since processed pseudogenes share 5′ and 3′ UTR sequences with their ancestral genes, they can exert regulatory control of parental gene expression by competing for the same miRNAs (49).

Recently, two HMGA1 non-coding processed pseudogenes, HMGA1P6 and HMGA1P7, have been identified and characterized. Both HMGA1P6 and HMGA1P7 have conserved seed matches for miRNAs targeting *HMGA1* and *HMGA2* genes and they work as competitive endogenous RNA (ceRNA), thereby protecting HMGAs from the inhibition of protein expression by miRNAs. Consistently, HMGA1 pseudogenes (Ps) also show oncogenic activity by inhibiting apoptosis and increasing cell proliferation and migration (50, 51). Moreover, a direct correlation between HMGA1 and HMGA1Ps expression in a set of human pituitary tumors, including somatotroph adenomas and NFPA or gonadotroph FSH-LH tumors has been found. Functional studies have also demonstrated that both pseudogenes affect pituitary cell proliferation and migration. Indeed, overexpression of HMGA1P6 or HMGA1P7 reduced the growth rate of the pituitary cell line AtT20 and, consistently with the role of HMGA1 in promoting cell migration, their enforced expression increases the migration of the same cells, thus indicating that HMGA1 pseudogene overexpression contributes to pituitary tumor development, thereby disclosing an additional mechanism responsible for the increased expression of HMGA1 in PAs (52).

A recent analysis of lncRNA expression in gonadotroph adenomas has unveiled a list of differentially regulated lncRNAs. Interestingly, among the most upregulated lncRNAs (fold-change 53,18 compared with normal pituitary) we found the RPSAP52 (ribosomal protein SA pseudogene 52) gene, which is the natural antisense of the *HMGA2* gene. It belongs to the category of the antisense lncRNAs, which are transcribed from the antisense strand and partially overlap with the coding strand, and thereby regulate expression of corresponding coding genes at transcriptional or post-transcriptional level through various mechanisms (53). Recent results demonstrate that the enforced expression of RPSAP52 is able to increase the HMGA2 protein level, induces cancer cell proliferation, and promotes cell cycle progression. Conversely, downregulation of HMGA2 protein and decreased proliferation rate of cancer cells were observed when RPSAP52 expression was inhibited by antisense oligonucleotides. Then, RPSAP52 is able to positively regulate the associated *HMGA2* gene, promoting its oncogenic activity, by mechanisms that are currently under investigation.

## Conclusion and Perspective

Taken together, the data overviewed here highlight the critical role of HMGA1 and HMGA2 overexpression in the onset of PAs. Moreover, the most recent studies evidence that aberrant HMGA protein levels in PAs are also sustained by epigenetic mechanisms. Indeed, it has been demonstrated that a set of miRNAs, constantly and drastically downregulated in PAs, are able to target both the *HMGA* genes. Interestingly, some of these miRNAs target also E2F1 whose activation, at least in animal models, is a critical step for PA development in transgenic mice overexpressing the *HMGA* genes.

**Figure 1:**
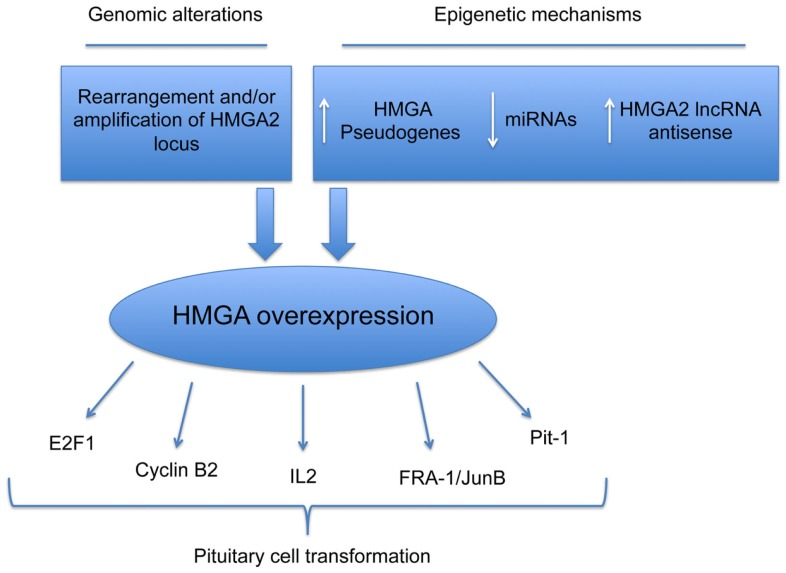
**Mechanisms responsible for HMGA overexpression in pituitary cell transformation**. Schematic representation of genetic and epigenetic mechanisms leading to overexpression of HMGA proteins in PAs.

Very recently, it has been shown that two HMGA1 pseudogenes are overexpressed in PAs. Their overexpression protects both the HMGA mRNAs from the miRNA-mediated downregulation of *HMGA* genes. Functional studies support the role of miRNA downregulation and HMGA1 pseudogene overexpression in pituitary tumorigenesis. In fact, miRNA-restoration reduces the proliferation rate of pituitary cell lines, with an increased G1 phase of cell cycle, accordingly with a reduction of the HMGA expression. Conversely, HMGA1 pseudogene overexpression, acting as ceRNA and protecting HMGAs from the inhibition of miRNAs, promotes the HMGA oncogenic activity. Finally, preliminary studies showed a drastic upregulation in PAs of the RPSAP52, the lncRNA antisense of HMGA2, which also leads to increased HMGA2 protein levels (Figure 1).

Then, all these data support an innovative therapy of the invasive PAs, based on the impairment of the HMGA protein function, that could not only be achieved directly, by the use of specific short interfering RNAs and/or drugs able to impair their activity, such as trabectedin (54), but also indirectly, restoring the expression of the HMGA-targeting miRNAs. Since miRNAs are able to target more than one gene that often code for proteins that are involved in the same or correlated pathways this last approach might represent an additional advantage for the antineoplastic therapy.

## Conflict of Interest Statement

The authors declare that the research was conducted in the absence of any commercial or financial relationships that could be construed as a potential conflict of interest. The Review Editor Rosa Marina Melillo declares that, despite being affiliated to the same institution as the authors Daniela D’Angelo, Francesco Esposito, and Alfredo Fusco, the review process was handled objectively and no conflict of interest exists.
